# Association between Perivascular Spaces and Progression of White Matter Hyperintensities in Lacunar Stroke Patients

**DOI:** 10.1371/journal.pone.0137323

**Published:** 2015-09-09

**Authors:** Caroline M. J. Loos, Pim Klarenbeek, Robert J. van Oostenbrugge, Julie Staals

**Affiliations:** 1 Department of Neurology, Maastricht University Medical Centre and Cardiovascular Research Institute Maastricht (CARIM), University Maastricht, Maastricht, The Netherlands; 2 Department of Neurology, Zuyderland Hospital, Heerlen, The Netherlands; University Medical Center (UMC) Utrecht, NETHERLANDS

## Abstract

**Objectives:**

Perivascular spaces are associated with MRI markers of cerebral small vessel disease, including white matter hyperintensities. Although perivascular spaces are considered to be an early MRI marker of cerebral small vessel disease, it is unknown whether they are associated with further progression of MRI markers, especially white matter hyperintensities. We determined the association between perivascular spaces and progression of white matter hyperintensities after 2-year follow-up in lacunar stroke patients.

**Methods:**

In 118 lacunar stroke patients we obtained brain MRI and 24-hour ambulatory blood pressure measurements at baseline, and a follow-up brain MRI 2 years later. We visually graded perivascular spaces and white matter hyperintensities at baseline. Progression of white matter hyperintensities was assessed using a visual white matter hyperintensity change scale. Associations with white matter hyperintensity progression were tested with binary logistic regression analysis.

**Results:**

Extensive basal ganglia perivascular spaces were associated with progression of white matter hyperintensities (OR 4.29; 95% CI: 1.28–14.32; p<0.05), after adjustment for age, gender, 24-hour blood pressure and vascular risk factors. This association lost significance after additional adjustment for baseline white matter hyperintensities. Centrum semiovale perivascular spaces were not associated with progression of white matter hyperintensities.

**Conclusions:**

Our study shows that extensive basal ganglia perivascular spaces are associated with progression of white matter hyperintensities in cerebral small vessel disease. However, this association was not independent of baseline white matter hyperintensities. Therefore, presence of white matter hyperintensities at baseline remains an important determinant of further progression of white matter hyperintensities in cerebral small vessel disease.

## Introduction

Perivascular spaces (PVS) are cerebrospinal fluid-filled spaces surrounding the small penetrating cerebral vessels in the basal ganglia and centrum semiovale [1]. Magnetic resonance imaging (MRI) visible PVS are associated with risk factors for cerebral small vessel disease (cSVD), such as age and hypertension [2,3]. They are also cross-sectionally associated with severity of other MRI markers of cSVD, especially white matter hyperintensities (WMH) [4]. Therefore, PVS are considered to be an early MRI marker of cSVD. The location of PVS may indicate different types of underlying cSVD: basal ganglia PVS are more strongly associated with blood pressure-related arteriopathy, whereas centrum semiovale PVS are associated with cerebral amyloid angiopathy [5]. Perivascular spaces in these two regions are also anatomically different: two leptomeningeal layers surround the basal ganglia small vessels, and superficial perforating small vessels in the centrum semiovale are only surrounded by one layer [2].

There is increasing evidence that blood-brain barrier (BBB) breakdown [4,6] is one of the primary steps in the pathogenesis of blood pressure-related cSVD. Derangement of the BBB leads to leakage of plasma components over the BBB into the vessel wall and perivascular space, and it is thought that this leads to enlargement of PVS [4,6]. Hypertension may cause BBB dysfunction through effects on the endothelium [7]. Previous studies have shown that elevated blood pressure (BP) levels are related with MRI markers of cSVD, including PVS and WMH [3,8–13], and that high BP levels are associated with WMH progression over time [14–15]. Furthermore, there is evidence that basal ganglia PVS are more strongly associated with hypertension and WMH than PVS in the centrum semiovale [4,16–17].

Although PVS are considered to be an early MRI marker of cSVD, it is unknown whether PVS, mainly in the basal ganglia, are related with progression of cSVD, and particularly with progression of WMH. Therefore, we aimed to determine the association between severity of PVS in the basal ganglia and centrum semiovale at baseline and progression of WMH after 2-year follow-up in lacunar stroke patients. We studied a patient cohort with highly prevalent MRI features of cSVD, namely lacunar stroke patients, and we included 24-hour BP levels in our analysis.

## Materials and Methods

### Study Population

From a lacunar stroke research project with first-ever lacunar stroke patients presenting at Maastricht University Medical Centre or Orbis Medical Centre Sittard, the Netherlands, between 2003 and 2008, we selected all first-ever lacunar stroke patients who had a baseline brain MRI and 24-hour ambulatory BP monitoring, and a two-year follow-up brain MRI [3, 12]. All patients participated with written informed consent in this research project, which has been approved by the local Medical Ethical Committee (Maastricht University Medical Centre). Lacunar stroke was defined as one of the recognized lacunar stroke syndromes [18] with a small (<2cm) lacunar lesion in the deep grey matter, pons or internal capsule, compatible with occlusion of a single deep perforating artery. In absence of such a lesion on baseline MRI, we used established clinical criteria for lacunar stroke syndromes [18]. Patients with potential cardioembolic sources or >50% carotid stenosis in at least one carotid artery were not included. Age, gender and vascular risk factors (hypertension, hypercholesterolemia, diabetes mellitus and smoking) were recorded as defined earlier [12].

### BP measurements

Ambulatory BP monitoring during a 24-hour period was performed after the acute stroke phase, between 1 and 6 months after stroke (mean 101±42 days). Patients continued their prescribed medication, and we registered the use of antihypertensive drugs. We calculated 24-hour ambulatory systolic blood pressure levels (24-h SBP) and 24-hour ambulatory diastolic blood pressure levels (24-h DBP). Details were described elsewhere [12].

### MRI scoring

Baseline MRI images (at 1,5 or 3T MRI scanner, Philips) were obtained as soon as possible and within 6 months after stroke onset. The MRI protocol consisted of axial T2-weighted fast spin echo and fluid attenuated inversion recovery (FLAIR) sequences, with slice thickness of 5 mm, gaps 0.5 mm and in-plane resolution 0.45 x 0.45 mm. The MRI protocol at two-year follow-up was similar to the baseline protocol. Two vascular neurologists independently assessed baseline and follow-up imaging.

**Fig 1:**
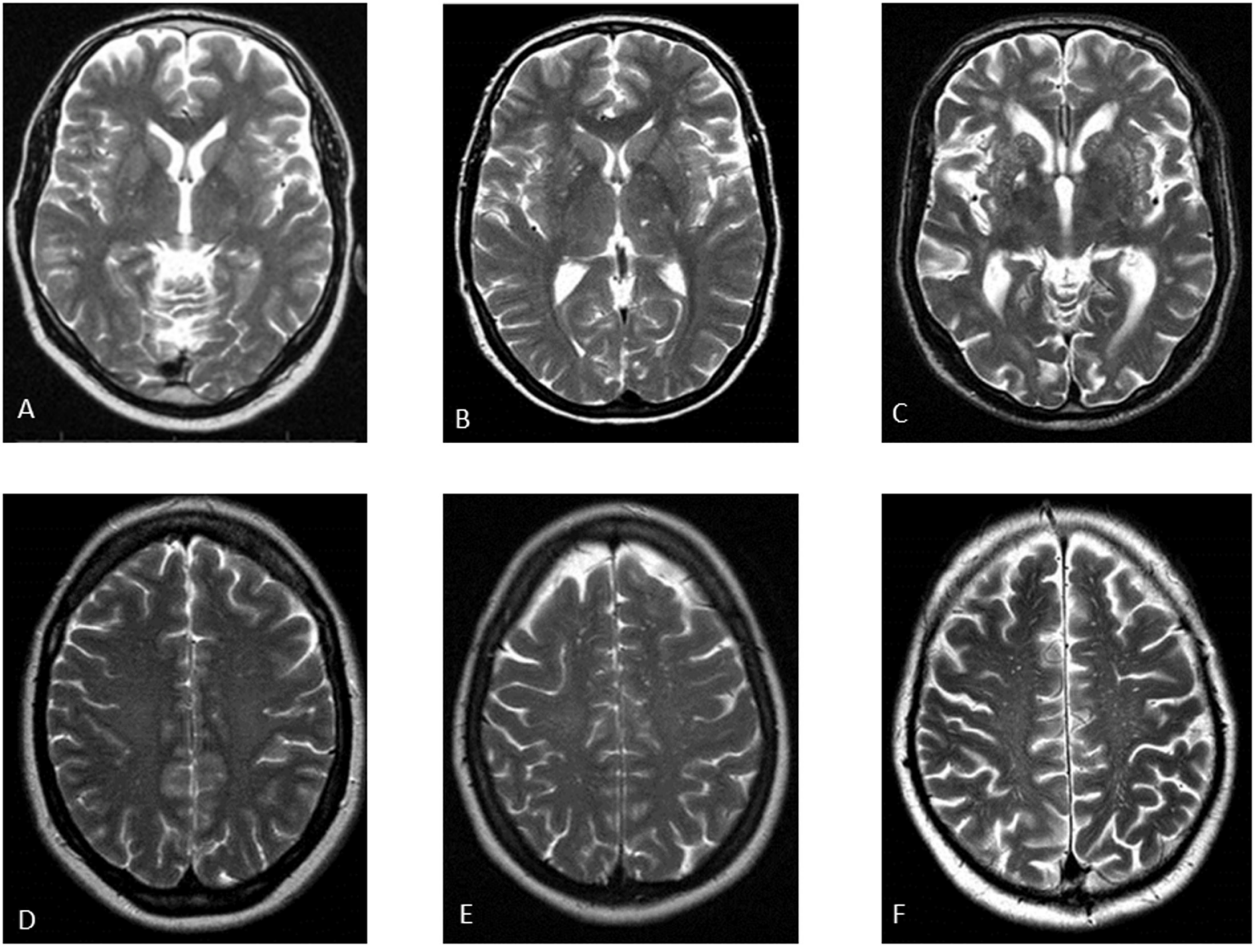
Examples of the categories of perivascular spaces by using a semi-quantitative three-category severity scale. (A-C): Perivascular spaces (PVS) in the basal ganglia; respectively none-to-mild, moderate and extensive. (D-F): PVS in the centrum semiovale; respectively none-to-mild, moderate and extensive.

Perivascular spaces (PVS) were defined as round, oval, or linear-shaped lesions with a smooth margin, absence of mass effect and with signal intensity equal to cerebrospinal fluid on T2-weighted images, and (if visible) hypointense on fluid–attenuated inversion recovery images without a hyperintense rim to distinguish them from old lacunar infarcts [1]. We distinguished PVS at the level of basal ganglia and centrum semiovale. We visually graded PVS on the slide with the highest number in one hemisphere, using a formerly described semi-quantitative three-category severity scale (none-to-mild, moderate and extensive) [3] (Fig 1). The inter-observer agreement was fair to good; weighted Cohen’s kappa 0.73 for basal ganglia PVS and 0.71 for centrum semiovale PVS [3]. We graded deep and periventricular WMH at baseline according to Fazekas’ scale [19]. The inter-observer agreement was good to excellent; weighted Cohen’s kappa 0.77 for periventricular WMH, and 0.84 for deep WMH [16].

At follow-up imaging, progression of WMH was assessed by a validated visual WMH change scale (The modified Rotterdam Progression Scale) [20]. This scale (range -7 to 7) measures a decrease, no change, or increase (-1,0,1 respectively) of WMH in three different periventricular and four different subcortical regions. We defined progression of WMH as an increase of WMH in one or more periventricular and/or subcortical regions. The inter-observer agreement was good; weighted Cohen’s kappa 0.79 for progression of WMH.

### Statistical analysis

Statistical analysis was performed using SPSS version 21.0 (Chicago, IL). Data are presented as n (%) for categorical variables or as mean ± standard deviation for parametric data. We determined the association between PVS categories and presence or absence of progression of WMH by binary logistic regression analysis. We corrected for age, gender, 24-hour SBP and 24-hour DBP. Next, we performed two models in which we additionally corrected for other vascular risk factors (including hypercholesterolemia, diabetes mellitus and smoking), or for baseline deep and periventricular WMH. Second, we also performed a binary logistic regression analysis to determine the possible relation between 24-hour ambulatory BP levels and progression of WMH. We performed two models in which we first corrected for age, gender and vascular risk factors (including hypercholesterolemia, diabetes mellitus and smoking), and second for age, gender and deep and periventricular WMH at baseline imaging. Statistical significance was considered at p<0.05. All relevant data are available in the Supporting Information files (data in S1 Dataset).

## Results

### Patients and Baseline Characteristics

Of 281 first-ever lacunar stroke patients who presented at Maastricht University Medical Centre, 35 patients were excluded because of presence of a carotid artery stenosis or possible cardioembolic source (most commonly atrial fibrillation). In total 116 patients refused to participate or had absolute contra-indications for MRI imaging. Of the remaining 130 patients, we excluded 16 patients because of an inadequate baseline MRI and/or inadequate BP monitoring data, leaving 114 patients at baseline. By applying the same inclusion and exclusion criteria we recruited 29 patients from Orbis Medical Centre Sittard (number and characteristics of non-included patients were not listed), which totals 143 included patients at baseline [3]. Patients were offered a clinical follow-up MRI after 2 years. Twenty patients were lost to follow up (5 patients died and 15 refused follow-up or could not be contacted), leaving 123 patients. To avoid overestimation of WMH progression, we excluded an additional 5 patients who had follow-up MRI at higher field strength than baseline MRI. Of the finally included 118 patients, 95 patients had baseline and follow-up MRI at 1.5 T (tesla), 20 patients had baseline MRI at 3.0 T with follow-up MRI at 1.5 T and 3 patients had baseline and follow-up MRI at 3.0 T.

Table 1 shows baseline patient characteristics of these 118 patients. Table 2 shows baseline and follow-up MRI characteristics. On baseline imaging, 34 (28%) patients had extensive basal ganglia PVS and 41 (35%) patients had extensive centrum semiovale PVS. On 2 year follow-up imaging, 54 (46%) patients had progression of WMH.

**Table 1:** Baseline patient characteristics.

	N = 118
Age at stroke onset (years), mean±SD	63±12
Male (%)	72 (61)
Hypertension (%)	79 (67)
Diabetes Mellitus (%)	16 (14)
Hypercholesterolemia (%)	93 (79)
Smoking (%)	49 (42)
24-h SBP (mmHg), mean±SD	139±18
24-h DBP (mmHg), mean±SD	83±12

Numbers (%) or means±SD (standard deviation); 24-h SBP: 24-hour ambulatory systolic blood pressure; 24-h DBP: 24-hour ambulatory diastolic blood pressure.

**Table 2:** MRI characteristics.

	N = 118
**Baseline MRI**	
*Periventricular WMH (%)*	
Fazekas grade 0	46 (39)
Fazekas grade 1	33 (28)
Fazekas grade 2	8 (7)
Fazekas grade 3	31 (26)
Fazekas grade 3	31 (26)
*Deep WMH (%)*	
Fazekas grade 0	33 (28)
Fazekas grade 1	50 (42)
Fazekas grade 2	14 (12)
Fazekas grade 3	21 (18)
*PVS at basal ganglia (%)*	
none-to-mild	42 (36)
moderate	42 (36)
extensive	34 (28)
*PVS at centrum semiovale (%)*	
none-to-mild	27 (23)
moderate	50 (42)
extensive	41 (35)
**Follow-up MRI**	
Progression of WMH (%)	54 (46)

WMH: white matter hyperintensities; PVS: perivascular spaces.

### Association between PVS and progression of WMH

Table 3 shows the association between baseline PVS and progression of WMH after 2 year of follow-up. Extensive basal ganglia PVS were associated with progression of WMH after 2 years (OR 5.23; 95% CI: 1.96–13.96; p<0.01). After adjusting for age, gender, 24-h SBP and 24-h DBP this association remained significant (OR 3.98; 95% CI: 1.23–12.88; p<0.05) and also with additional adjustment for other vascular risk factors (OR 4.29; 95% CI: 1.28–14.32; p<0.05). However, with additional adjustment for periventricular and deep WMH at baseline, the association between extensive PVS and WMH progression lost statistical significance (OR 1.49; 95% CI: 0.37–6.07; p = 0.58). No association was found between centrum semiovale PVS and WMH progression.

**Table 3:** Association between baseline PVS and progression of WMH by binary logistic regression analysis.

Progression of WMH				
OR (95% CI)				
	Unadjusted	Model 1	Model 2	Model 3
**Basal ganglia PVS**				
Non to mild	1.00	1.00	1.00	1.00
Moderate	2.07 (0.84–5.10)	1.85 (0.67–5.11)	1.70 (0.60–4.83)	1.22 (0.37–3.99)
Extensive	5.23 (1.96–13.96) **	3.98 (1.23–12.88)*	4.29 (1.28–14.32) *	1.49 (0.37–6.07)
**Centrum semiovale PVS**				
Non to mild	1.00	1.00	1.00	1.00
Moderate	0.89 (0.34–2.32)	0.99 (0.36–2.75)	0.82 (0.29–2.37)	1.15 (0.34–3.88)
Extensive	2.05 (0.77–5.51)	2.05 (0.72–5.87)	1.88 (0.65–5.49)	2.42 (0.64–9.14)

Model 1 adjusted for age, gender, 24-h systolic blood pressure (24-h SBP) and 24-h diastolic blood pressure (24-h DBP). Model 2 adjusted for age, gender, 24-h SBP, 24-h DBP and vascular risk factors (diabetes mellitus, hypercholesterolemia and smoking). Model 3 adjusted for age, gender, 24-h SBP, 24-h DBP and baseline white matter hyperintensities (WMH). OR: odds ratio; CI: confidence interval; PVS: perivascular spaces.

*p<0.05;

**p<0.01.

### Association between BP and progression of WMH

Table 4 shows the associations between baseline 24-hour ambulatory BP levels and progression of WMH after 2 year of follow-up. We did not find a significant association between baseline 24-h SBP and progression of WMH after 2 years. Twenty-four hour DBP levels seemed to be negatively associated with progression of WMH (OR 0.83; 95% CI: 0.49–0.96; p<0.05). However, this association lost significance after adjustment for age, gender, vascular risk factors and baseline WMH.

**Table 4:** Association between baseline ambulatory 24-hour blood pressure levels and progression of WMH by binary logistic regression analysis.

Progression of WMH			
OR (95% CI)			
	Unadjusted	Model 1	Model 2
**ambulatory 24-h BP (mmHg)**			
SBP	0.86 (0.69–1.06)	0.80 (0.63–1.02)	0.78 (0.59–1.02)
DBP	0.83 (0.49–0.96)*	0.89 (0.55–1.14)	0.81 (0.43–1.04)

Results binary logistic regression analysis presented as OR per 10 mmHg increase in systolic blood pressure (SBP) or 5 mmHg in diastolic blood pressure (DBP). Model 1 adjusted for age, gender and vascular risk factors (diabetes mellitus, hypercholesterolemia and smoking). Model 2 adjusted for age, gender and baseline white matter hyperintensities. OR: odds ratio; CI: confidence interval; WMH: white matter hyperintensities; 24-h BP: 24-hour ambulatory blood pressure;

*p<0.05.

## Discussion

Our study shows that extensive basal ganglia PVS are associated with progression of WMH over two years of follow-up, independent of age, gender and vascular risk factors. However, this association was not independent of the presence of WMH at baseline. We did not find a positive association between centrum semiovale PVS and WMH progression and we did not find an association between 24-h SBP or 24-h DBP levels at baseline and WMH progression.

Studies with a cross sectional design showed that PVS, and in particular basal ganglia PVS, are associated with severity of other MRI markers of cSVD, including WMH [3–4,16–17] and with increased BBB permeability [21]. Hypertension could lead to endothelial dysfunction and BBB breakdown [2,7], which are considered to be the primary steps in the pathogenesis of cSVD [4,6]. Leakage of plasma components over the BBB leads to damage of the cerebral small vessel wall and enlargement of PVS [6]. Therefore, as PVS seem to appear early in the course of cSVD, the amount of PVS might be an early MRI marker of brain damage related to cSVD. In CADASIL (cerebral autosomal dominant arteriopathy with subcortical infarcts and leukoencephalopathy), a genetic form of cSVD, severity of temporal PVS is strongly related with total WMH volume [22] and PVS in the temporal lobes have a pathological correlation with temporal WMH [23]. Our study shows that extensive basal ganglia PVS are associated with progression of WMH in lacunar stroke patients. Even though our results need to be confirmed in other cohorts, we suggest that basal ganglia PVS might be an early marker for progression of WMH in blood pressure-related cSVD, although the presence of WMH still remains most important for further progression.

It has been suggested that WMH tend to form around PVS [6], however we did not find an association between PVS in the white matter of the centrum semiovale and progression of WMH. However, we tested the association between overall severity of centrum semiovale PVS and general progression of WMH, which does not correlate them spatially. Another explanation could be that severity of centrum semiovale PVS is underestimated in those cases with overshadowing presence of extensive WMH. Finally, PVS in the centrum semiovale may have a different pathogenesis than basal ganglia PVS. They may be linked to cerebral amyloid angiopathy rather than blood pressure-related cSVD [5,24].

We did not find a positive association between 24-hour ambulatory BP levels and progression of WMH after 2 years. Even though several other studies showed that high BP levels are associated with WMH progression [14–15], this was not found by all [25]. The relationship between WMH and BP remains complex [2]. Previous studies have shown that cumulative, prior elevated BP levels are more associated with WMH compared to concurrent BP [26–28]. A J-curved relation between BP and WMH has also been shown [29]. We do not have data on severity and duration of hypertension, nor on previous treatment of elevated BP levels in our patients, and this may be important in the association between BP and progression of WMH. Furthermore, a substantial portion of our patients had anti-hypertensive treatment during the follow-up period and this may have influenced WMH progression. There is evidence that patients with untreated and uncontrolled hypertension have greater progression of WMH compared to patients with controlled or treated BP levels [15,30].

Our study has several limitations. First, not all patients were scanned at the same MRI field strength, which might have led to bias in grading lesions. However, analysis including only patients with the same field strength at baseline and follow up (n = 98) did not change the results of our main analysis (results not shown). Second, we measured PVS and progression of WMH by using visual scales and not quantitative volumetric techniques. However, a quantitative method for counting PVS does not exist and visual semi-quantitative assessment is still the reference-standard method [1]. The modified Rotterdam Progression Scale is the most reliable visual assessment tool for WMH progression and correlates well with volumetrics [20,31]. Third, a cohort of lacunar stroke patients with advanced cSVD and a substantial amount of WMH at baseline, might not be ideal to test for an association between severity of PVS at baseline and progression of WMH. To confirm the predictive role of PVS as an early MRI marker of WMH progression, we suggest a study in a population at risk and in an early stage of cSVD (for example hypertensive patients), with a long follow-up time.

In conclusion, extensive basal ganglia PVS are associated with WMH progression after 2 year follow-up in lacunar stoke patients. However, this association was not independent of WMH at baseline. Therefore, baseline WMH still remain an important determinant of progression of WMH in cSVD.

## Supporting Information

S1 Dataset (XLSX)
